# Tissue Specific Origin, Development, and Pathological Perspectives of Pericytes

**DOI:** 10.3389/fcvm.2018.00078

**Published:** 2018-06-27

**Authors:** Tomoko Yamazaki, Yoh-suke Mukouyama

**Affiliations:** ^1^Laboratory of Stem Cell and Neuro-Vascular Biology, Genetics and Developmental Biology Center, National Heart, Lung, and Blood Institute, Bethesda, MD, United States; ^2^Robert W. Franz Cancer Center, Providence Portland Medical Center, Earle A. Chiles Research Institute, Portland, OR, United States

**Keywords:** pericyte, development, differentiation capacity, origin, heterogeneity

## Abstract

Pericytes are mural cells surrounding blood vessels, adjacent to endothelial cells. Pericytes play critical roles in maturation and maintenance of vascular branching morphogenesis. In the central nervous system (CNS), pericytes are necessary for the formation and regulation of the blood-brain barrier (BBB) and pericyte deficiency accompanies CNS diseases including multiple sclerosis, diabetic retinopathy, neonatal intraventricular hemorrhage, and neurodegenerative disorders. Despite the importance of pericytes, their developmental origins and phenotypic diversity remain incompletely understood. Pericytes express multiple markers and the origin of pericytes differs by tissue, which may cause difficulty for the identification and understanding of the ontogeny of pericytes. Also, pericytes have the potential to give rise to different tissues *in vitro* but this is not clear *in vivo*. These studies indicate that pericytes are heterogeneous in a tissue- and context- dependent manner. This short review focuses on recent studies about identification of pericytes, heterogeneous origin of pericytes during development and in adults, and the differentiation capacity of pericytes, and pericytes in pathological settings.

## Introduction

Pericytes are mural cells surrounding blood vessels and embedded within the basement membrane of the vasculature and adjacent to endothelial cells (1). Pericytes cover microvessels such as arterioles, venules and capillaries, while large-diameter vessels like arteries and veins are covered by vascular smooth muscle cells (VSMCs), the other type of mural cells. Mural cells are known to play fundamental roles in vascular network formation: pericyte coverage is critical for vascular stability and structure. In the central nervous system (CNS), pericytes play an essential role in the maturation and maintenance of the blood brain barrier (BBB) (2–4), which is established by the interaction between the microvessels (endothelial cells and pericytes) and surrounding astrocytes within the neurovascular unit. The BBB is a diffusion barrier which blocks the inflow of various molecules and toxins from blood to brain, but not all CNS vessels equally contribute to the BBB: several areas of the brain including the pituitary gland, pineal gland, subventricular zones and choroid plexus are not protected by the BBB. The diversity of the BBB architecture and function remains largely unexplored. Given that pericyte density, morphology, and function vary in different vascular beds (5), these observations may reflect their heterogeneous characteristics. In this review, we give an overview of pericytes with a focus on their heterogeneity. A more objective definition of different subsets of pericytes may help to shed light on novel therapeutic approaches for neuro-vascular diseases associated with pericyte loss such as stroke and Alzheimer's disease.

## Identification of pericytes

**Figure 1:**
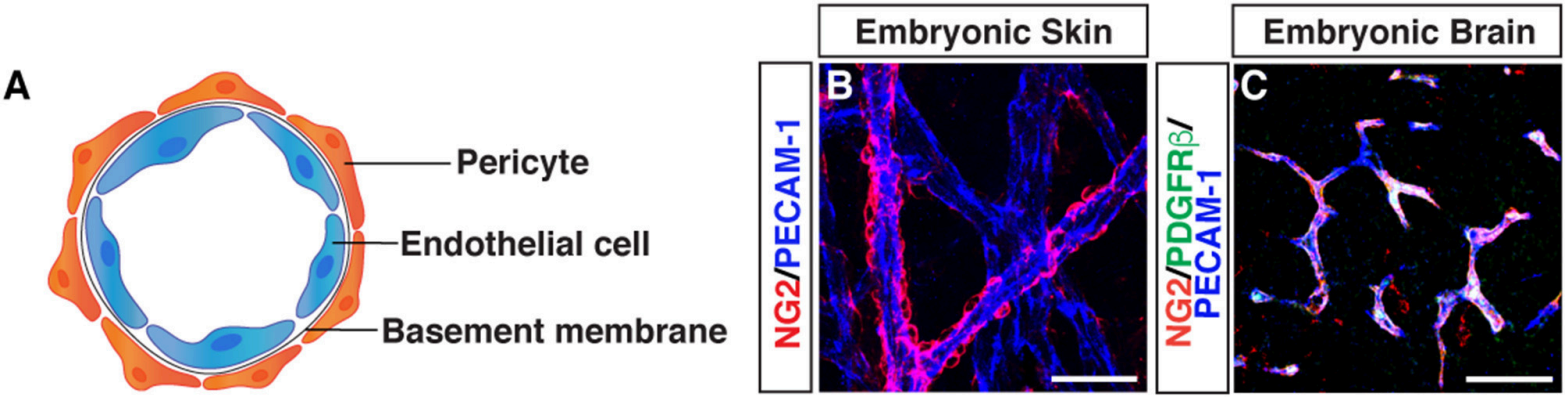
Morphology of pericytes. **(A)** Schematic image of pericyte-endothelial cell interaction. **(B)** Whole-mount immunostaining of mouse embryonic skin with antibodies to pericytes (NG2, red) and endothelial cells (PECAM-1, blue) at embryonic day (E) 15.5. Pericytes are semicircular shape and cover blood vessels. **(C)** Triple immunostaining of E15.5 mouse embryonic brain with antibodies to pericytes (NG2, red; PDGFRβ, green) and endothelial cells (PECAM-1, blue). Scale bars, 50 μm.

Morphological characteristics of pericytes were defined by electron microscopy: pericytes possess a cell body with a prominent nucleus and contain a small amount of cytoplasm with several long processes covering the endothelial wall. Pericytes are embedded in the basement membrane where they interact with endothelial cells: pericyte-endothelial cell interaction enhances basement membrane assembly (Figure 1).

Although the ultrastructural characteristics of pericytes has been well studied using electron microscopy, pericytes remain a relatively poorly defined cell type, without highly specific markers available for their identification (6). The following markers have been used as pericyte markers, but no general pan-pericyte molecular marker has been discovered because of their heterogeneous distributions and functions in various tissues (2, 5, 7). We should note that these markers are also expressed by other cell types including perivascular fibroblasts, VSMCs and macrophages. Chondroitin sulfate proteoglycan4/neural glial antigen 2 (NG2) is expressed on the surface of pericytes during angiogenesis (8, 9), but is also expressed on glial precursor O2A cells in the CNS, which generate either oligodendrocytes or astrocytes *in vitro*. Platelet-derived growth factor receptor beta (PDGFRβ) (10, 11) is one of the most widely studied molecular marker expressed in pericytes. PDGF-B/PDGFRβ signaling is essential for pericyte proliferation and recruitment to blood vessels. Alpha smooth muscle actin (αSMA) (12), desmin (13), and vimentin (14) are contractile filaments. Regulator of G protein signaling 5 (RGS5) (15, 16) is a GTPase activating protein for Giα and Gqα, and the expression pattern of RGS5 overlaps with expression pattern of NG2 and PDGFRβ. CD146, also known as melanoma cell adhesion molecule (MCAM) has been used as a marker for pericytes and VSMCs as well as endothelial cells (17). Recent studies suggest that CD146 regulates PDGFRβ activation and involves in BBB integrity (18) and is essential for pericyte recruitment (19). CD13/aminopeptidase N (APN) (20) is a membrane-bound metalloprotease which was originally identified as a myeloid cell marker (21). CD13 has been used as a surface marker for brain pericytes (22, 23). Overall, current immunohistochemical approaches to identify pericytes use antibodies against these proteins, depending on tissue and microvessel types. The expression of these markers varies depending on developmental stages, organs, pathological situations, and *in vitro* or *in vivo* conditions. Therefore, anatomical characteristics combined with at least two molecular markers are important to define pericytes. Indeed, recent studies have revealed that double *PDGFR*β-*EGFP* and *NG2-DsRed* fluorescence reporter expression patterns are valuable for pericyte/mural cell identification of the CNS tissues (24, 25). Although the expression of αSMA is known as a marker for both pericytes and VSMCs, capillary pericytes do not express αSMA (12): capillary pericytes in embryonic skin express only NG2 and PDGFRβ but not αSMA (26). In contrast, capillary pericytes in tumors do express αSMA (27).

Genetic mouse models including transgenic markers, fluorescent reporters and lineage tracing lines are valuable tools to trace the pericyte lineage during development and in pathological conditions: nuclear β-galactosidase reporter (28) as well as emerging fluorescent reporter and lineage tracing lines using the promoter of *PDGFR*β (24, 29)*, NG2* (24, 30, 31)*, or Tbx18* (32) are available. Alternatively, a fluorescent Nissl dye specifically labels brain pericytes and enables the imaging in the live mouse (33).

## Developmental origin of pericytes

The developmental origin of pericytes is heterogeneous, and much remains to be deciphered. Most commonly described, and best understood is their origin from mesenchymal stem cells (34). Chick-quail chimera analysis (35, 36) and genetic lineage tracing experiments using neural crest-specific *Cre* recombinase lines such as *Wnt-1-Cre* and *Sox10-Cre* mice in combination with a *Cre*-mediated reporter line demonstrate that neural crest contributes to pericytes in the face, brain, and thymus (36–40). Using similar genetic lineage tracing experiments, the origin of pericytes in the gut (41), lung (42), and liver (43) in mice has been traced to the mesothelium, a single layer of squamous epithelium. In the heart, the epicardial mesothelium gives rise to coronary pericytes and VSMCs (44–46). Recent studies have demonstrated that some endocardium also contributes to coronary pericytes (47). These studies clearly indicate that the origin of pericytes is heterogeneous in a tissue- and context- dependent manner.

**Figure 2:**
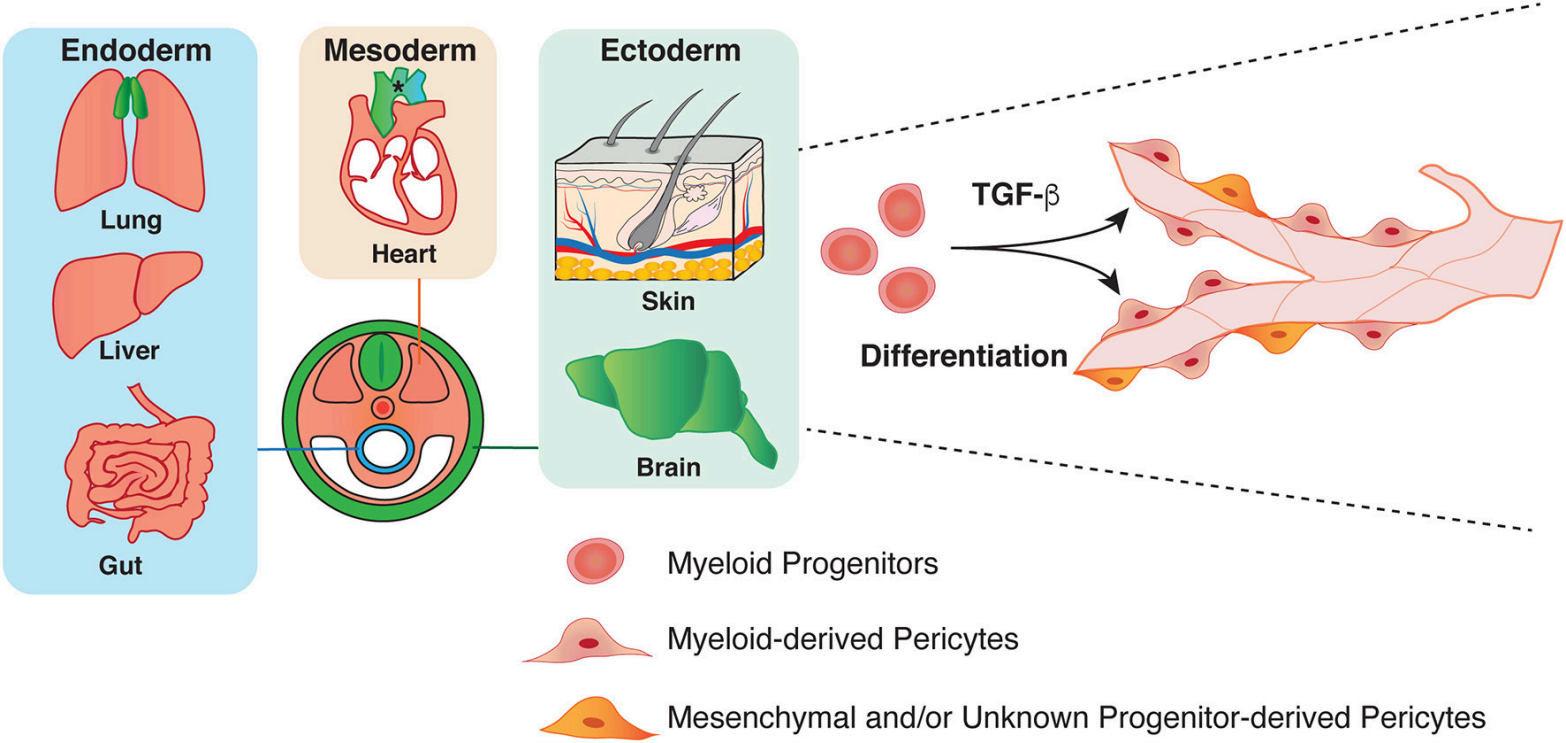
Heterogeneity in the origin of pericytes. Pericytes in both endoderm and mesoderm-derived organs, such as lung, liver, gut, and heart have mesoderm origin (orange). Pericytes in the aorta region have several origins: secondary heart field, neural crest, and somite (asterisk). Previous studies have demonstrated that ectoderm-derived neural crest give rise to pericytes in brain and thymus (green). Our group has recently shown that mesoderm-derived myeloid progenitor cells differentiate into pericytes in ectoderm-derived organs such as skin and brain through TGF-β signaling during development.

We have recently revealed that myeloid progenitor cells differentiate into a subset of pericytes in the ectoderm-derived skin and brain during development (Figure 2) (26). Using high-resolution whole-mount imaging and a series of genetic lineage tracing experiments with hematopoietic cell-specific *Vav-Cre* and myeloid cell-specific *CD11b-Cre* lines in combination with a *Cre*-mediated fluorescent reporter line, we found that the developmental sources of pericytes are heterogeneous and some pericytes are derived from myeloid progenitors in the developing skin and brain. Mutant mice lacking myeloid lineage exhibit defective pericyte development. Moreover, TGF-β promotes the differentiation of myeloid progenitors into pericytes *in vitro* and *in vivo*. In a similar line of research, some CD31^+^ F4/80^+^ macrophages contribute to cerebrovascular pericytes during embryogenesis (48). Insight into the heterogeneous origins of pericytes will have important implications for understanding the establishment of the organ-specific vascular networks during embryonic angiogenesis. Moreover, whether pericytes of different origins have different functions in these tissues remains to be elucidated.

It is an intriguing question of whether pericytes of heterogeneous origins at embryonic stages remain in adult: one possible scenario is that a subtype of pericytes may become dominant in adult. Moreover, adult tissue-resident progenitors contribute to pericytes in pathological situations. Indeed, mesenchymal stem cells generate pericytes after radiation therapy (49), while mesenchymal tumors such as bone and soft tissue sarcomas can be derived from pericytes (50). Glioblastoma stem cells generate pericytes to support tumor growth (51). Defining the origin of adult tissue pericytes needs the development of a cell-type specific inducible *Cre* recombinase line such as *CreER*. Transient *Cre* activity produces fluorescent reporter positive cells at a defined developmental time point and then allows for the tracking of their progeny in adults. The inducible lineage tracing experiment also allows us to examine whether pericytes of different origins could differentially contribute to the neovascularization processes in pathological conditions such as tumor angiogenesis and wound healing.

## Pericytes in adult and diseases

Pericytes have been reported as a component of stem cell niches and mesenchymal stem cells. In the bone marrow, NG2^+^ pericytes in arterioles promote hematopoietic stem cell (HSC) quiescence and are important for HSC maintenance (52). Likewise, NG2^+^/Nestin^+^ pericytes associate with portal blood vessels in fetal liver and are required for the HSC niche (53). In the adult brain, neural stem cells (NSCs) located in the largest germinal region of the forebrain, the ventricular-subventricular zone (V-SVZ). Endothelial cells and pericytes in the V-SVZ form the NSC niche, and V-SVZ pericytes secrete diffusible factors that increase the proliferation and enhance neuronal differentiation (22). *In vitro* differentiation capacity of pericytes into mesenchymal cell types (e.g., adipocytes, chondrocytes, osteoblasts, fibroblasts, VSMCs) has been proven in a multitude of studies (54–58). Pericytes facilitate repair process after myocardial infarction in the heart (59, 60), while pericytes regenerate injured and dystrophic skeletal muscles (61). *In vivo* lineage tracing experiments have reported pericytes as progenitors of white adipocytes (62), follicular dendritic cells (63), odontoblasts (mesenchyme-derived dentine producing cells) (64), and skeletal muscle (61). Differentiation capacities of pericytes into neurons, astrocytes, and oligodendrocytes have been also reported (56). In addition, pericytes have been reported to play a major role as fibroblast progenitors in fibrotic responses (65).

Cardiac pericytes have been studied as a therapeutic target after injury. Cardiac pericytes account for up to 5% of the total non-cardiomyocyte cell population (66). Recent studies identified microvascular pericytes in the human ventricular myocardium and demonstrated that human cardiac pericytes express mesenchymal stem/stromal cell markers including CD44, CD73, CD90, and CD105 (58). Indeed, cardiac pericytes have the capacity to differentiate into mesodermal lineage: osteo-, chondro-, and adipogenesis, but no potential for skeletal myogenesis *in vitro* (58). Many cardiac diseases are associated with fibrosis, an accumulation of fibroblasts and an excess of extracellular matrix proteins which may affect the architecture and function of the organ or the tissue. Pericytes have been shown to contribute to fibrosis organ-dependently (67): NG2^+^/Nestin^−^ type 1 pericytes are recruited and accumulated in the ischemic interstitial space around fibrotic tissue but do not contribute to fibrosis (67).

However, a series of lineage tracing experiments with *Tbx18CreERT2* line, which is selectively expressed by pericytes and VSMCs in multiple adult organs, has revealed that adult pericytes of heart, brain, skeletal muscle and fat depots do not behave as multipotent progenitors in aging and different pathological situations such as a high-fat diet and injury. In addition, pericytes do not contribute to fibroblasts in fibrotic responses (32). These results are in contrast to the previous studies that demonstrated the multipotent potential of pericytes. The discrepancies might be explained by the differing methods used in the studies to identify pericytes as well as the different behavior of *in vitro* and *in vivo* pericytes. The population of pericytes which can differentiate into mesenchymal cell types may vary by organ and developmental stage *in vivo*. It is also possible that pericyte differentiation requires stem cell-like cells of non-pericyte origin (64).

## Conclusion

The absence of any single marker to identify pericytes complicates the study of the ontogeny and the differentiation capacity of pericytes. Much remains to be elucidated regarding how and when the origin of pericytes in an organ is determined and whether pericytes have potential for differentiation *in vivo*. There are questions about how pericytes derived from different origins behave when they co-exist in a tissue: do they show identical gene expression patterning and behavior in adult tissues under normal and pathological conditions? Future studies using pericyte-specific lineage tracing mice and precise gene-expression profiling will pave the way for the understanding of the spatial-temporal pericyte behavior *in vivo* and may contribute to new therapeutic strategies for diseases associated with pericyte loss and/or dysfunction.

## Author contributions

Both authors listed have made a substantial, direct and intellectual contribution to the work, and approved it for publication.

### Conflict of interest statement

The authors declare that the research was conducted in the absence of any commercial or financial relationships that could be construed as a potential conflict of interest.
